# Light thinning can improve soil water availability and water holding capacity of plantations in alpine mountains

**DOI:** 10.3389/fpls.2022.1032057

**Published:** 2022-10-12

**Authors:** Yuan Gao, Zhibin He, Xi Zhu, Longfei Chen, Jun Du

**Affiliations:** ^1^ Linze Inland River Basin Research Station, Chinese Ecosystem Research Network, Key Laboratory of Eco-Hydrology of Inland River Basin, Northwest Institute of Eco-Environment and Resources, Chinese Academy of Sciences, Lanzhou, China; ^2^ University of Chinese Academy of Sciences, Beijing, China

**Keywords:** *P. crassifolia*, soil water characteristic curve, thinning intensity, soil water holding capacity, soil water availability

## Abstract

The establishment of large-scale forest plantations in the arid and semi-arid area of the Qilian Mountains in China has effectively protected water and soil resources and enhanced carbon sequestration capacity of forest ecosystems. However, the effects of different management practices in these plantations on soil water holding capacity (SWHC) and soil water availability (SWA) are uncertain in this fragile ecosystem. Here, we investigated the effects of no thinning (NT), light thinning (LT, 20% thinning intensity), and heavy thinning (HT, 40% thinning intensity) on SWHC and SWA in different soil depths of a forest plantation, and compared them to those in a natural *Picea crassifolia* forest (NF). Our results revealed that at low soil water suction stage, SWHC in the plantations (LT, HT, and NT) was greater in the topsoil layer (0-40 cm) than that in the NF site, while SWHC in the subsoil layer (40-80 cm) in NF was significantly greater than that in the thinning stands. At medium and high-water suction stage, SWHC in LT and NF stands was greater than that in HT and NT. Soil water characteristic curves fitted by VG model showed that the relative change in soil water content in LT topsoil layer was the smallest and SWHC was greatest. Changes in soil physicochemical properties included higher bulk density and lower total porosity, which reduced the number of macropores in the soil and affected SWHC. The bulk density, total porosity, silt content, and field capacity were the main factors jointly affecting SWA. High planting density was the main reason for the low SWA and SWHC in NT, but this can be alleviated by stand thinning. Overall, 20% thinning intensity (light intensity thinning) may be an effective forest management practice to optimize SWHC and SWA in *P. crassifolia* plantations to alleviate soil water deficits.

## Introduction

To cope with global warming, China has embarked on CO_2_ emission reduction goals of “double carbon” (carbon peaking and carbon neutrality) (Shan et al., 2022). Biological carbon sequestration into forests, grasslands, lakes is an important means of increasing CO_2_ absorption and alleviating greenhouse gas concentration. Among them, forest restoration and afforestation represent a significant carbon sink, which is further characterized by its low cost, large size, and a high ecological added value (Veldkamp et al., 2020).

In the arid and semi-arid areas of northwest China, afforestation was accomplished through a series of ecological projects including the Three-North Shelterbelt System, the Natural Forests Protection and Restoration Project, and Returning Farmland to Forests and Grasslands. The area of forest land has increased by 3.35 × 10^7^ ha since the 1980s, of which for 42.5% is due to afforestation (Chen et al., 2019). The Qilian Mountains is a source of several large inland rivers, these ecological projects contributed 85.4% of additional plantation lands in the Qilian Mountains, and enhanced the water conservation capacity of forest ecosystem in this area. Afforestation also effectively promoted a substantial increase in forest stock and carbon sink and made positive contributions to the mitigation of global warming and improvement in the regional natural ecosystems (Zhao et al., 2021).

Soil moisture is a key factor of water and energy exchange in the Soil-Plant-Atmosphere Continuum (Bogena et al., 2013). Soil water holding capacity (SWHC) is the ability of soil to hold water and is an important indicator of soil water dynamics (Hollis et al., 2015). Due to the influence of soil parent material, topography, land use type, soil salinity, and other factors, SWHC status exhibits high spatial-temporal heterogeneity (Schrumpf et al., 2011; Mohammadi and Meskini-Vishkaee, 2013; Korres et al., 2015; Knighton et al., 2019). Soil water availability (SWA) is a measure of soil water that can be effectively used by vegetation. It is defined as the soil water content between filed capacity and permanent wilting point and is the basis for evaluating the construction of ecological vegetation and the restoration of forest ecosystem in water-scarce areas. Therefore, it is of great practical significance to investigate and improve the SWA and SWHC of cultivated vegetation in the arid and semi-arid regions where the overall ecological vulnerability and vegetation carrying capacity are low.

Thinning and understory vegetation removal in forests are widely used silvicultural strategies in forest management (Baena et al., 2013; Chase et al., 2016) to improve the availability of growth-limiting resources (light, water, nutrients, etc.) for residual trees by adjusting stand density and vegetation structure. However, numerous studies have reported that changes in forest canopy structure and land-use patterns caused by afforestation and tree stand thinning may lead to profound changes in soil dynamic properties, and directly or indirectly affect many soil functional processes (Guo and Gifford, 2002; He et al., 2013; Kaarakka et al., 2014; He et al., 2018; Kurniawan et al., 2018). On the one hand, thinning reduces forest canopy density and rain interception, affecting soil moisture status and topsoil temperature in forests, and leading to increased soil water evaporation and reduced soil water content at topsoil (Schrumpf et al., 2011; He et al., 2014; Sohn et al., 2016; Del Campo et al., 2019). On the other hand, soil compaction caused by human disturbance (grazing) leads to an increase in soil bulk density, a decrease in soil organic carbon storage, soil infiltration, water storage capacity (Zhu et al., 2015; He et al., 2018; Hong et al., 2020; Veldkamp et al., 2020). These influences may accelerate the formation of surface runoff, leading to increased soil erosion. Therefore, it is necessary to obtain data support from tree stand thinning experiments in different site conditions and soil depths to extend our scientific knowledge of thinning consequences.

The Qilian Mountains (QLM) is located in the key area of ecological security strategy of China (Wang et al., 2019). It is an important ecological water conservation area in the Yellow River Basin and the core area for the construction of ecological barriers in the Qinghai-Tibet Plateau. Since the establishment of the Qilian Mountains National Nature Reserve in the late 1980s, the forest plantation area in QLM increased from 4.36 × 10^5^ ha in 1989 to 8 × 10^5^ ha in 2020, an 45.5% increase. The reserve plays a key role in maintaining regional ecological stability and species diversity. *Picea crassifolia*, as a dominant tree species in the QLM, is mainly distributed in Gansu, Qinghai, Ningxia, and Inner Mongolia of China (Chang et al., 2017). It prefers cold and humid environments, and often forms large pure forests on shaded and semi-shaded slopes of the subalpine zone with an altitude of 2250-3450 m and average precipitation of 290-520 mm (Gao et al., 2021). Because of its strong drought resistance, it has become an important afforestation tree species in eastern Qinghai province and the QLM.

However, due to insufficient understanding of the water demand of vegetation, the extent of local water resources, and water consumption law of vegetation. Ecological restoration projects in these water-scarce areas are limited by single-structure plantations and high-density afforestation (Garcia-Salgado et al., 2018). Furthermore, low forest productivity, soil degradation, and poor stability of the ecosystem are also common problems in these *P. crassifolia* plantation forests (He et al., 2018). To alleviate the soil drought caused by soil water deficit and improve the stem-level productivity. Keeping the stability of the plantation ecosystem and the sustainability of its ecological benefits in the QLM. Timber in the form of whole-tree harvested has been an important and common forest management measure for *P. crassifolia* plantation in this area. However, the response of changes in soil hydrology and hydraulic characteristics to the silvicultural practice of *P. crassifolia* plantation and natural forests at different soil depths is poorly known. In the process of vegetation restoration and management, understanding the SWHC and SWA under different thinning intensities is of great significance for selecting sustainable stand planting density in semi-arid areas. Thus, in this study, our objectives were to determine (1) the effects of different thinning intensities on SWHC and water availability, and (2) the main factors affecting SWHC and soil water availability.

## Materials and methods

### Site description

**Figure 1:**
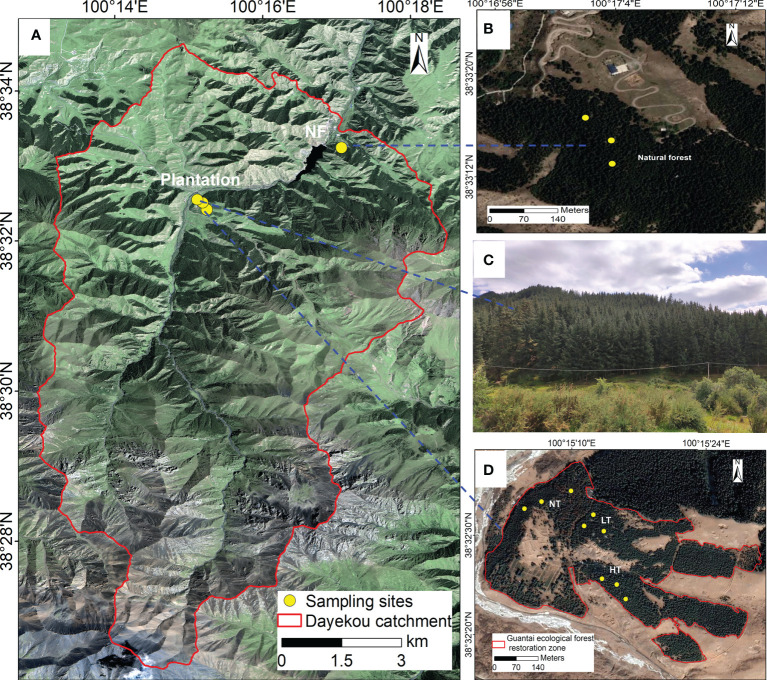
Geographical location of Dayekou watershed and sampling sites **(A)**. Sampling sites **(B, D)** and plantation landscape **(C)** with differently-managed of *P. crassifolia*.

The study area was located in the Guantai Ecological Forest Restoration Zone (38°41′N, 100°19′E) of Xishui Natural Reserve, Qilian Mountains National Park, in northwest China. The area has a typical cold temperature and semi-arid climate, with a mean annual temperature of about 2.5C and mean annual precipitation of about 385 mm (from 1994 to 2015) (Chen et al., 2021). About 80% of the precipitation is concentrated in the growing season from May to September. Terrain, precipitation, and temperature differences significantly affect the distribution patterns of native vegetation, which is represented by a mosaic of forests, grasslands, and small areas of shrublands (Gao et al., 2021). Grey cinnamon and chestnut soils are the common soil types in this area. It is dominated by loam and sandy loam. pH is 7.23-8.5, which is alkaline soil. The dominant tree species *Picea crassifolia* is found mainly on shady and semi-shady slopes at an altitude of 2250-3450 m, often forming large areas of forests. *Berberis kansuensis* and *Potentilla glabra* are common shrub species in the understory layer. Herbaceous species, such as *Carex moorcroftii*, *Elymus cylindricus*, *Iris lactea*, and *Stipa capillata* are mainly found on sunny and semi-sunny slopes. In the early 1980s, part of the grassland on the semi-shady slopes of this catchment was converted to a *P. crassifolia* plantation (Zhu et al., 2017). Stand density in the recent afforestation areas was about 4600 trees ha^-1^ (**Figure 1**). In 1997, 1998, and the spring of 2006, to avoid the interference and compaction of vehicles and heavy machinery on the soil, a manual chain saw was used for thinning and harvesting the artificial forests in the study area to varying degrees to remove trees with poor growth, and severely affected by plant diseases and insect pests among the forest stand.

### Experimental design, soil sampling, and determination of soil properties

In the summer of 2019, we conducted a field survey of *P. crassifolia* plantation and of a nearby natural secondary forests (NF). We determined tree ages to be 54 years (natural forest) and 37 years (plantation forest) by interviewing local herders and landowners, and by drilling core samples in several trees. We selected stands with similar altitude, aspect, and other site conditions for our study **Table 1**). Stands were subject to four different management practices (**Figure 1**, **Table 1**), including (i) no thinning, no human disturbance in the natural forest (reference stand), with 1652 trees ha^-1^ (NF), (ii) no thinning in the plantation (NT), with 4458 trees ha^-1^, (iii) light thinning in the plantation (LT) which involved the removal of 20% of stems and final density of 3558 trees ha^-1^, (iv) heavy thinning in the plantation (HT) with the removal of 40% of stems and final density of 2721 trees ha^-1^. Detailed information on these sites is given in **Table 1**. We established three replicate plots of 30 × 30 m in each of the four management practices for a total of 12 plots. The distance between each plot was at least 30 m to avoid edge effects (He et al., 2018). Tree height, stand density, and DBH were measured in each plot (**Table 1**). In addition, we used the global positioning system (GPS) to accurately determine geographic coordinates and altitude of each site.

**Table 1:** Site conditions and stand characteristics of the study area. Values (±SE) represent the means ± standard error, and the sample size (n) is 3, followed by different lowercase letters within rows indicate significant differences at *P*＜0.05.

Forest types	MS^a^	Elevation (m)	Slope (°)	Aspect (°)	Stand density (trees ha^-1^)	Plantation age (yr)	DHB^b^ (cm)	Height (m)
Natural forest	NF	2779	14.6	327NW	1652±95	54	12.6±0.16c	8.9±1.03c
Plantation	NT	2761	12.3	331NW	4458±41	38	9.9±0.16a	7.2±1.21a
	LT	2796	11.8	324NW	3558±52	37	10.2±0.13b	7.4±1.32b
	HT	2817	10.9	340NW	2721±55	36	10.6±0.16c	7.8±1.48b

a MS represents the management practices of P. crassifolia. NF, NT, LT, and HT represent forest stands with natural forest (reference stand), no thinning, light thinning (20% thinning intensity), and heavy thinning (40% thinning intensity), respectively.

b DHB is the diameter at breast height (1.3 m above ground).

In each plot, 12 soil profiles with the dimension of 1.0 × 1.0 × 1.0 m were excavated (after removing the surface litter layer). Undisturbed soil samples were collected from 0-10, 10-20, 20-40, 40-60, and 60-80 cm soil depth using a ring cutter with a fixed volume of 100 cm^3^. Within each soil profile, three replicates for each soil layer and 15 soil samples were taken in total. Meanwhile, at least 500 g of soil was collected from each layer of all soil profiles and stored in self-sealing bags to determine soil particle-size distribution, organic carbon, and other indicators. Soil samples in ring cutters were taken to the laboratory for total wet weight measurements and the weight of soil sample marked as W_1_. Soil samples were immersed in ultrapure water for at least 12 hours, with care being taken that the upper edge of the ring cutter was not submerged. Samples were weighed again when soil was completely saturated and marked as W_2_. Then, soil samples were placed in a container, covered with fine sand for 72 hours and weighed and marked as W_3_. Finally, samples with ring cutters were dried to a constant weight in an oven at 108C for at least 12 hours and marked as W_4_. The calculation formula for soil properties is as follows (Zhang et al., 2021):


(1) $$BD=(W_{4}-W_{0})/V$$

(2) $$TP=(W_{2}-W_{4})/V\times 100\%$$

(3) $$FC=(W_{3}-W_{4})/W_{4}\times 100\%$$

Where BD, TP, and FC represent bulk density (g/cm^3^), soil total porosity (%), and field capacity (%), respectively. W_0_ and V denote the weight and volume of the empty ring cutter, respectively. W_2_, W_3_, and W_4_ represent the weight of soil sample after saturation, 72 hours, and drying, respectively.

Self-sealing bags of soil samples were placed in a ventilated, cool, and sun-free area to air dry, then ground and screened using a 2 mm mesh. Soil particle-size distribution was measured using a laser granulometer (Mastersizer 3000E, UK). Soil organic carbon (SOC) was measured using colorimetry after digestion with sulfuric acid and potassium dichromate at 5% concentration (Robinson, 1994). Soil pH value was determined with a pH meter (RS232, AZ8601 pH acidometer, China) in a 1:5 ratio of soil-water mixture (Chen et al., 2021). The Kjeldahl procedure was used to determine soil total nitrogen (TN) (Bradstreet, 1954).

### Soil water characteristic curve

Soil water characteristic curve is the relationship between soil water content and soil water suction, which is used to determine SWHC (Hollis et al., 2015), and it is typically established with the centrifuge method. The principle is to set the corresponding to centrifugal speed and time according to different soil water suction (negative pressure of soil water) (Zhang et al., 2019). Then, when soil water suction pressure and centrifugal force reach equilibrium, weighted of the dehydrated soil sample at specific water suction. Then the volumetric water content corresponding to each soil water suction is calculated using mass water content and bulk density, so as to determine the water characteristic curve for undisturbed soil samples (Van Genuchten, 1980).

The soil water characteristic curve in unsaturated soil is mainly determined by a large number of laboratory and field tests (Van Genuchten, 1980; Van Genuchten and Nielsen, 1985). To accurately fit the soil water characteristic curve and obtain unsaturated hydraulic conductivity and diffusivity on this basis. It is necessary to fit the measured data of the soil water characteristic curve, so the selected fitting model must be able to fully describe the relationship between soil water content and soil matrix potential. For this reason, Van Genuchten proposed an equation to describe the relationship between pressure head (h) and water content (θ) in 1980 through a large number of indoor and outdoor experiments to measure relevant parameters, that is, the soil water characteristic curve of vadose zone. In this study, we used the common Van Genuchten model (VG model for short) to fit the soil water characteristic curve (Van Genuchten, 1980). The VG model equation is as follows:


(4) $$\frac{\theta -\theta_{r}}{\theta_{s}-\theta_{r}}={\left[\frac{1}{1+\alpha |\psi_{m}{|}^{n}}\right]}^{m}$$

Where *θ*, *θ_s_*, *θ_r_* denote soil water content (%), saturated soil water content (%), and residual soil water content (%), respectively. *Ψ_m_* is soil matric potential (negative of soil water suction, bar). A (*α*), *m*, and *n* are all fitting parameters (*m* = 1-1/*n*), where *α* denotes the reciprocal of the soil air intake value, *m* and *n* are morphological parameters (estimated from observed soil water retention data) of the water characteristic curve. FC and wilting coefficient (WC) can be estimated at 0.33 bar and 15 bar, respectively (Tamea et al., 2009).

For this study, the ring cutter soil sample was infiltrated for 24 hours until saturated and weighed to determine saturated water content. Then, a dehydration experiment was carried out with a high-speed centrifuge (Kokusana H-1400PF, Japan) at a temperature ≤ 26C (Wen et al., 2021). The centrifuge speed was set at 190, 430, 600, 860, 1210, 1480, 1710, 1920, 2700, 4280, 6060, and 7420 r/min in 12 gradients, corresponding to 0.01, 0.05, 0.1, 0.2, 0.4, 0.6, 0.8, 1, 2, 5, 10, and 15 bar soil water suction. After each centrifuge operation, the experimental soil sample was taken out and weighed. In the end, soil water content and corresponding water suction of each soil sample were calculated using the drying method.

**Figure 2:**
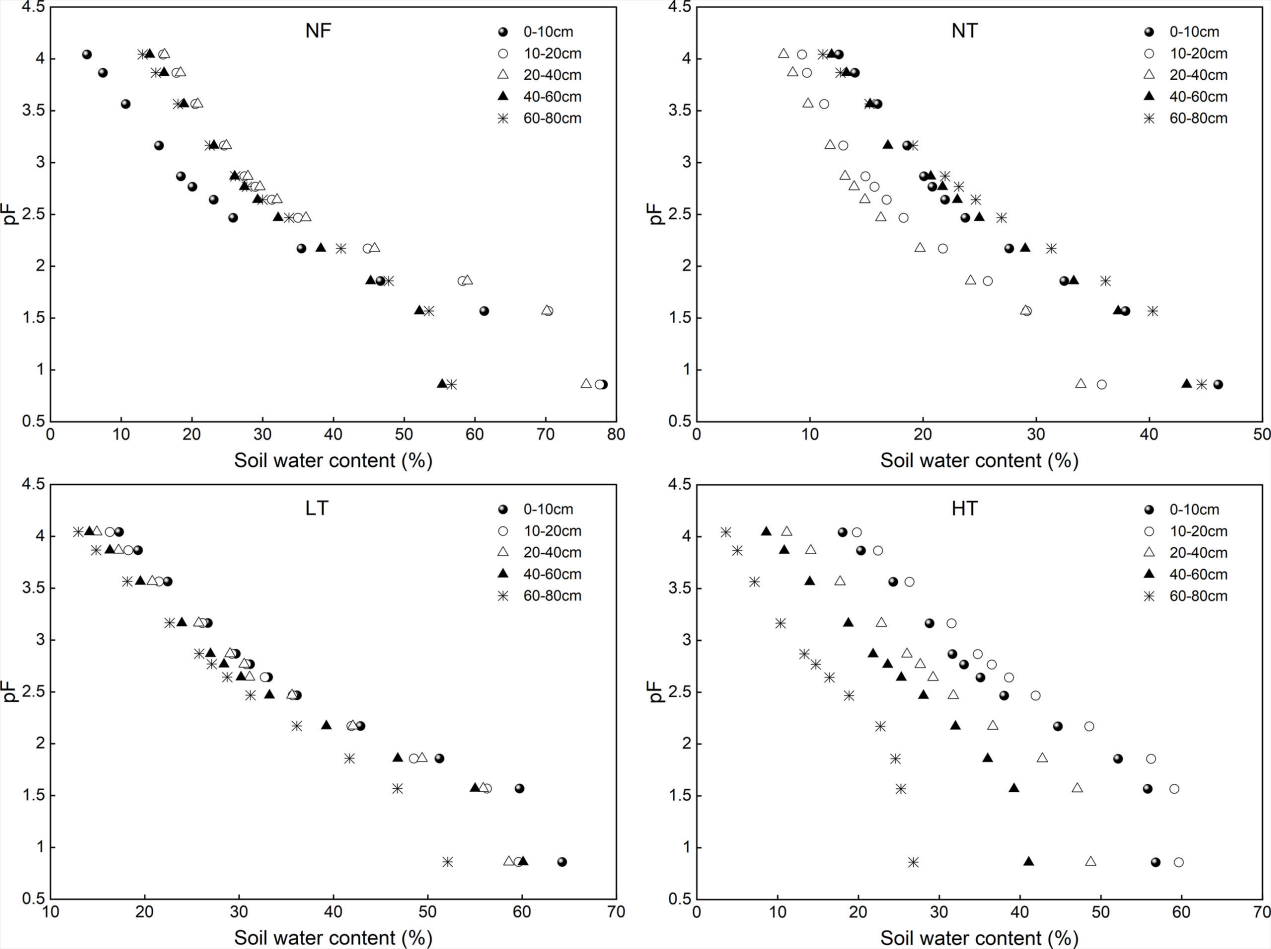
Soil water characteristic curves for different soil layers under different management practices of *P. crassifolia*.

To express changes in soil water characteristic curve under different management practices more easily, we adopted the ordinary logarithm of the absolute value of the matric potential (pF) to express soil water suction (Cianfrani et al., 2019); to do this, we took the logarithm of the absolute value in centimeters of the soil water suction column as the vertical axis and soil water content as the horizontal axis to draw the soil water characteristic curve (Schofield and Botelho, 1938) (**Figure 2**). Based on soil moisture, soil water suction was divided into three stages: 0< pF ≤ 1.86, 1.86< pF ≤ 2.52, and pF > 2.52 (Cianfrani et al., 2019). Among them, 0< pF ≤ 1.86 was the stage of low water suction and the soil water characteristic curve was relatively scattered; 1.86< pF ≤ 2.52 was the transition from low to medium-high water suction, and soil water content decreased significantly; pF > 2.52 was the high water suction, and the curves were relatively parallel.

### Demarcation of soil water availability

Field capacity is usually regarded as the dividing point between gravity water and available water, that is, the upper limit of available water (Ritchie, 1981). Employing the above equation (4), we determined that 0.33 bar of water suction was the FC and 15 bar was the WC. Based on the division scheme of soil available water developed by Zhang et al. (1994), we defined SWA under different soil water suction at three levels: rapidly-available water (RAW) (-0.33 bar ~ -10 bar), slowly-available water (SAW) (-10 bar ~ -20 bar), and unavailable water (UAW) (< -20 bar). The total available water (TAW) was the sum of RAW and SAW, that is, soil available water.

### Statistical analysis

The differences among forest stands in soil physical and chemical properties (TN, TP, and SOC) due to different management practices were analyzed with one-way analysis of variance (ANOVA) and the least significant difference test (LSD) (P< 0.05). Pearson correlation analysis was used to determine the relationships between SWA and other factors, such as BD, WC, FC, pH, TP, SOC, and soil particle-size distribution. Statistical analyses were conducted using SPSS software version 27.0 (SPSS Inc., Chicago, IL, USA, 2020).

## Results

### Effects of different stand thinning intensities on soil water characteristic curve

**Figure 3:**
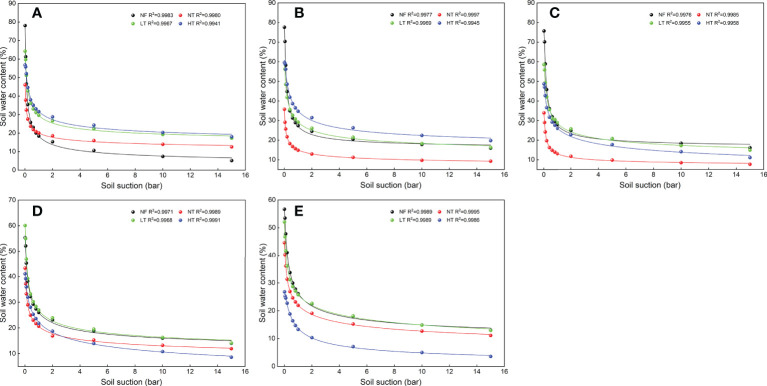
Soil water characteristic curves fitting with the Van Genuchten model for different soil depths under different management practices of *P. crassifolia*. Labels **(A–E)** indicate different soil depths, namely 0-10cm, 10-20cm, 20-40cm, 40-60cm, and 60-80cm.

The soil water characteristic curves for each soil depth under different stand management practices were almost parallel to each other, and the overall shape was “S”. Different characteristics were obtained for SWHC (**Figure 2**). The faster the soil water content decreased, the lower the SWHC of the stand. With an increase in soil water suction, SWHC differed significantly for different soil layers and management practices (**Figures 2**,**3**). In general, SWHC in LT, HT, and NF was higher than that in NT at low water suction (0< pF ≤ 1.86) at 0-60 cm soil depth, and low at medium and high water suction (1.86< pF ≤ 2.52 and pF > 2.52). At 60-80 cm depth, SWHC in HT and NF stands was higher than that in LT and NT stands at low water suction. However, SWHC in NF at medium and high-water suction was higher than that in LT and HT plots at 0-60 cm soil depth.

The fitting of soil water characteristic curves for each soil depth (0-10, 10-20, 20-40, 40-60, and 60-80 cm) under different management practices are shown in **Figure 3**. The coefficient of determination *R^2^* between the measured data and the soil water characteristic curves fitted with the VG model was > 0.99. As a whole, with an increase in soil water suction, soil water content in all soil layers showed a decreasing trend. Within a soil water suction range of 0-2 bars, soil water characteristic curves decreased quickly. Soil water content for all soil depths decreased slowly under different stand management practices when soil water suction was 2-10 bars, and it intended to be stable when soil water suction exceeded 10 bars.

### Soil water availability

**Figure 4:**
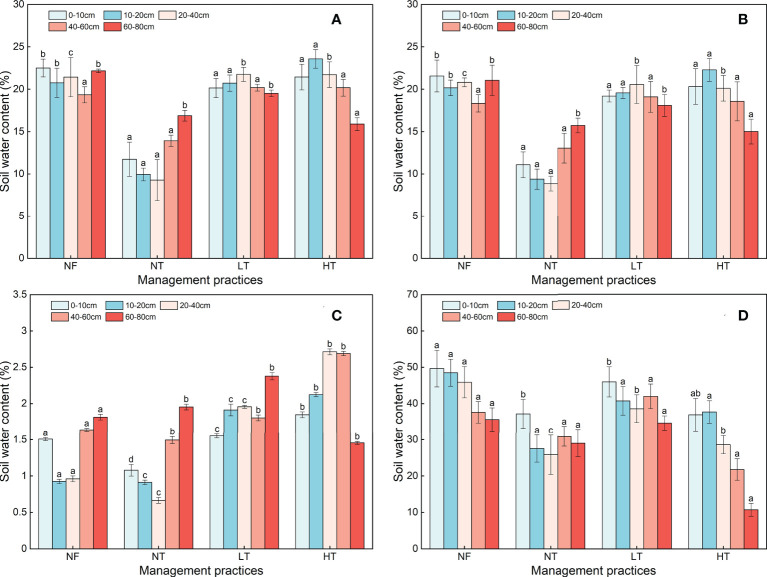
Total available water (TAW, **A**), rapidly-available water (RAW, **B**), slowly-available water (SAW, **C**), and unavailable water (UAW, **D**) for each soil depth under different management practices. Different lowercase letters above the column indicate significant differences among the soil depths at the same management practice (P < 0.05).

At the soil depth of 0-40 cm, TAW content in NT was significantly lower than that in NF, and there was an apparent dry layer at 20-40 cm soil depth, which was not significantly different than that in LT and HT (*P<* 0.05) (**Figure 4**). The TAW in the 40-60 cm soil layer decreased with an increase in thinning intensity at first, then increased, and finally stabilized. The NT plot had the lowest TAW; there was no significant difference in TAW between HT and NF plots (*P<* 0.05). In the subsoil at 60-80 cm, TAW under the different management practices gradually decreased with an increase in thinning intensity, and there was no difference in any of the plots (*P<* 0.05). The variability characteristics of RAW at 0-40, 40-60, and 60-80 cm depths were similar to those of TAW (**Figure 4**). The available water content in the 0-80 cm soil layer range from 9.28 to 23.57%, and the range of variation in TAW in each soil layer was smallest in LT, followed by NF and HT, and finally NT.

Slowly-available water of *P. crassifolia* forest under the different management practices decreased first and then increased with increasing soil depths in all but the 60-80 cm in HT plot. Further, SAW in the 0-40 cm soil layer in NT was significantly lower than that in HT, LT, and NF (*P<* 0.05). However, UAW content in the 0-60 cm soil layer in HT was significantly higher than that in other plots. The UAW content of other *P. crassifolia* forest plots decreased with an increase in soil depth except for 40-60 cm in NT and LT stands, with the greatest decrease in HT, and smallest in NT.

### Soil physicochemical properties related to SWA

**Figure 5:**
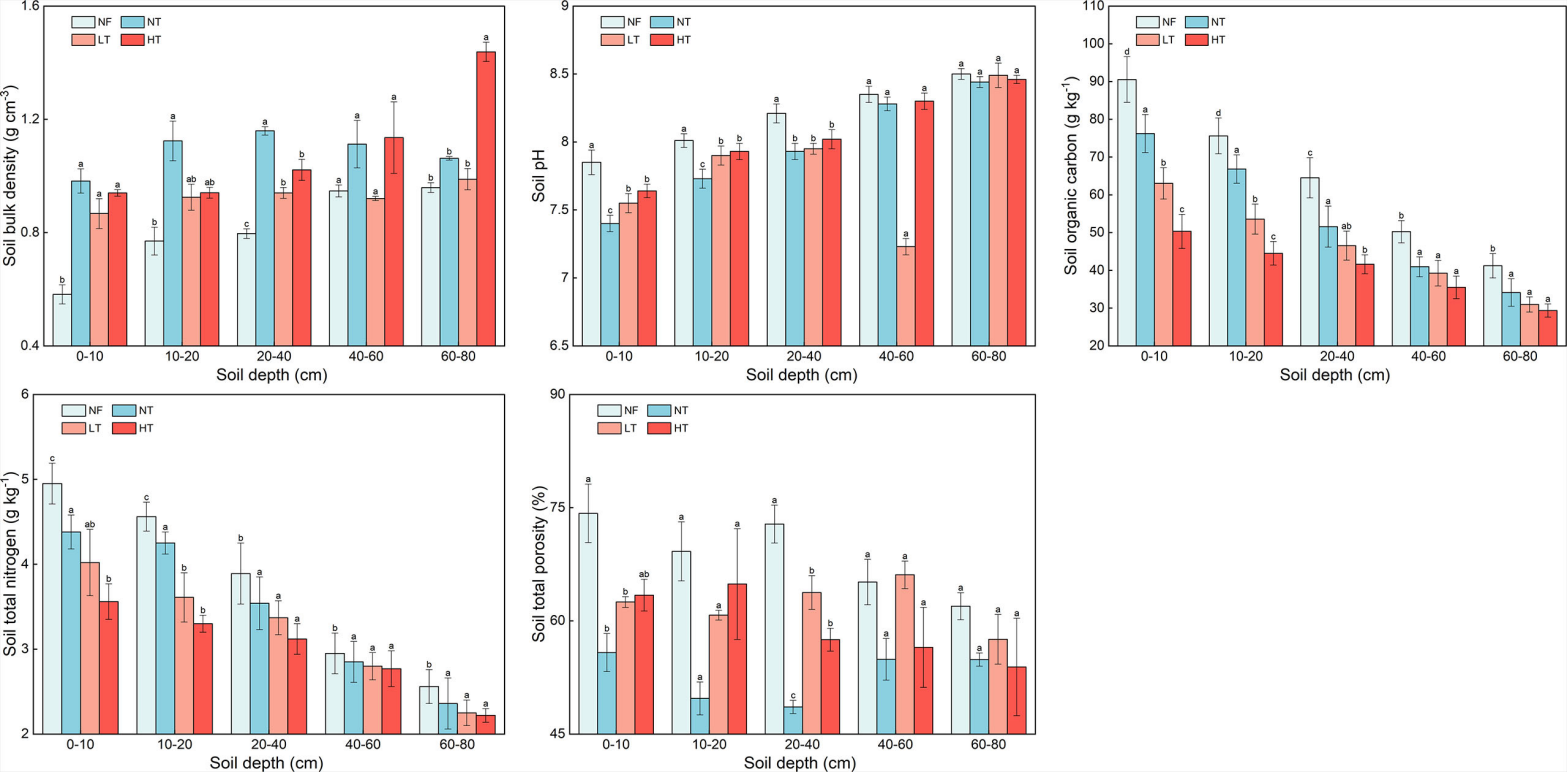
Comparison of soil bulk density (BD), pH, soil organic carbon (SOC), soil total nitrogen (TN), and soil total porosity (TP) among different management practices and soil layers. Different lowercase letters above the column indicate significant differences among the thinning treatments at the same soil depth (P < 0.05).

Soil BD, pH, SOC, TN, and TP at different soil depths under different management practices are shown in **Figure 5**. Generally, BD tended to increase with an increase in thinning intensity and soil depth except for 40-80 cm soil in NT; BD was highest in HT at 60-80 cm at 1.43 g cm^-3^. There were significant differences in BD among NF, NT, and HT (*P<* 0.05). At 0-40 cm depth, BD in NT was significantly higher than that in other stands, with the highest BD at 20-40 cm at 1.16 g cm^-3^, and lowest at 0-10 cm in NF at 0.58 g cm^-3^.

Soil pH in *P. crassifolia* increased slowly with an increase in soil depth in all stands, except for the 40-60 cm depth in LT stand, but the differences with depth were not significant (*P<* 0.05). Both SOC and TN exhibited a decreasing trend with an increase in soil depth, and the decrease at the topsoil (0-40 cm) was greater than that at subsoil (40-80 cm). The decrease in SOC at topsoil layer of in NT was the greatest at 32.3%, and that at subsoil layer was the lowest at 16.6%. The decrease in TN at topsoil layer of the NF stand was the greatest at 21.4%, and at subsoil layer was the lowest at 13.2%. On the whole, TP fluctuated and decreased with an increase in soil depth. The variation tendency of TP in NF and HT plots was similar, while TP in LT did not vary with depth (*P<* 0.05) and that in NT was lowest.

Soil texture in the *P. crassifolia* forest in the study area was mainly loam and sandy loam (**Supplementary Figure 1**, **Supplementary Figure 2**). Sand content was highest at 51.9%, followed by silt at 46.4%, and clay at 11.7%. Generally, clay content in NF, NT, and LT at 0-80 cm soil depth increased with thinning intensity, while that in HT decreased with depth and then increased, with the lowest at 9.2% at 10-20 cm. In addition, clay content in each soil layer in LT was significantly higher than that in other sample plots. The silt content in NF decreased with soil depth, while that in NT was higher in subsoil (40-80 cm) than in topsoil. In contrast, sand content in NF increased with soil depth, while it noticeably decreased at 40-80 cm in NT.

### Relationships between SWA and soil properties

**Figure 6:**
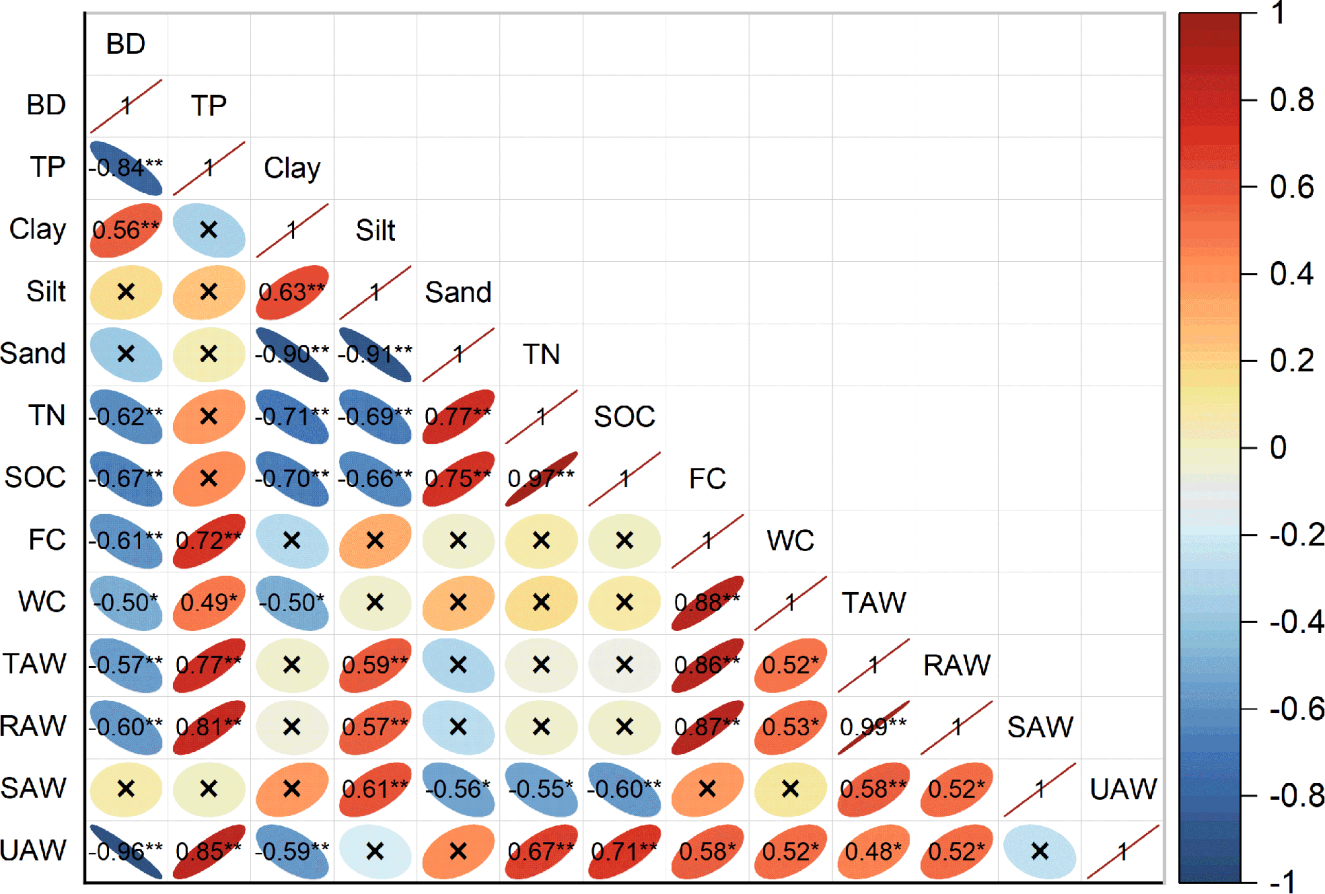
Pearson correlation analysis between soil water availability (SWA) and soil physicochemical properties, nutrient characteristics, and other parameters. Significance denoted by *P < 0.05 and **P < 0.01.

We found that SAW was closely related to BD, TP, silt content, and FC (**Figure 6**). Soil BD at 0-80 cm depth was significantly negative correlated with TP, TN, SOC, and FC (*P*< 0.01), and significantly positive correlated with clay content (*P<* 0.01). Both TAW and RAW were significantly negative correlated with BD (*P*< 0.01), significantly positive correlated with TP, silt content, and FC (*P*< 0.01), and positive correlated with WC (*P<* 0.05). SAW and silt content were significantly positive correlated with TAW (*P*< 0.01), significantly negative correlated with SOC (*P*< 0.01), and negative correlated with sand content and TN (*P*< 0.05).

## Discussion

### Effects of different forest management practices on SWHC

The soil water characteristic curve is the relationship between soil water content and matric potential under an unsaturated state. The soil matrix is a medium for maintaining and conducting materials (water, air, and solute) and energy (heat). Its function is determined by soil properties and soil porosity. Therefore, different stand management practices may lead to changes in SWHC by affecting soil bulk density, soil texture, and porosity. Soils with high clay contents have high soil water content at any level of soil water suction (Yao et al., 2014). In this study, clay content at 0-80 cm soil depth in the LT plot was higher than that in NT, HT, and NF, the sand content was lowest, and SWHC was highest (**Supplementary Figure 2**). Sand content was higher for all soil depths in NF, and all depths except 60-80 cm in NT, and their SWHC was lower than in LT and HT stands (**Figure 3**, **Supplementary Figure 2**). This is mainly due to a larger number of fine pores and greater surface energy in high-clay than in low-clay soils, which can absorb and maintain more water. Soil with relatively more sand and less clay has more macropores from which soil water is easily lost, resulting in low SWHC.

He et al. (2018) and Hong et al. (2018) observed that thinning and afforestation significantly decreased soil carbon stock at 0-70 cm soil depth, while pH value showed an increasing trend in *P. crassifolia*, *Pinus sylvestris* var. *mongolica* and *Pinus tabuliformis* plantations. We found a similar phenomenon in this study. A change in BD can cause a change in TP, which in turn changes the size and distribution of soil porosity. Human disturbance and soil compaction were aggravated with an increase in thinning intensity, stand structure and environmental factors have changed significantly, resulting in the increase of BD. The pores formed by large soil particles in the soil will be gradually filled and compacted by small soil particles, reducing the TP and inevitably affecting soil water characteristic curves (Sanchez, 2019). Therefore, soil saturated water content decreased with an increase in BD, and the variability in soil water characteristic curve was not significant at the stage of higher soil water suction (**Figures 3**, **5**). In the 0-5 bar range of soil water suction, soil water holding or releasing capacity mainly depends on the number and distribution of macropores. As soil water suction increases, water in macropores can be easily discharged. Therefore, the downward trend in soil water characteristic curve is significant. Water retained in small pores drains slowly, so the soil water characteristic curve tends to be flat (**Figure 5**). This finding was similar to that in a study by Ozcan et al. (2013) and Zhang et al. (2021), where the higher the TP and the lower the BD, the stronger the water holding capacity.

### Factors regulating SWA in *P. crassifolia* forests

Soil water availability is the main index for evaluating the degree of soil water utilization by plants and the effect of water stress on plant growth (Cianfrani et al., 2019). It is also an important limiting factor in afforestation in arid and semi-arid areas (Zhou and Li, 1995). Our correlation analysis showed that soil properties (BD, TP, and silt content), and FC were the main factors affecting SWA under different management practices (**Supplementary Figure 2**, **Figure 6**, **Supplementary Figure 3**). With an increase in thinning intensity, soil exposure, and compaction, soil surface structure was increasingly disturbed, BD increased. In this way, the topsoil is hardened and porosity is decreased. Soil drainage and infiltration weakened. Resulting in reduced in SWA.

It is generally accepted that the content of clay and sand in the soil can significantly affect its soil water status (Yao et al., 2014; Minasny and McBratney, 2018; Zagyvai-Kiss et al., 2019). However, we found different results. **Figure 6** and Supplementary **Figure 3** showed that TAW was significantly positively correlated with silt content (*P*< 0.01), but it had little relationship with clay or sand content. The sand content in each soil layer accounted for the largest proportion (43.7-67.4%) with the increase in thinning intensity, but it had a negative correlation with SWA. Although soil layers did not differ in clay contents, fluctuating from 8 to 15.8%, a small change would significantly affect soil porosity. The higher content of silt and clay in LT than in NF and NT increased the number of fine pores in the soil and effectively promoted SWA.

Changes in soil properties caused by thinning can indirectly affect FC and WC. In general, with the increase in thinning intensity, FC in NF, LT, and HT exhibited a decreasing trend, while FC in NT first decreased first (0-40 cm) and then increased (40-80 cm) (**Table 2**). FC and WC under different management practices decreased in the order of HT > NT > LT > NF, and FC in each soil layer in LT was the closest to FC in NF. This was consistent with the results of Ma et al. (2017).

**Table 2:** Field capacity and wilting coefficient of *P. crassifolia* under different management practices.

Soil depths(cm)	Management Practices								
	NF(FC)	NF (WC)	NT(FC)	NT(WC)	LT(FC)	LT(WC)	HT(FC)	HT(WC)	AVE
0-10	29.16	6.66	25.07	13.33	38.62	18.48	40.80	19.36	23.19
10-20	38.33	17.57	19.24	9.29	37.90	17.18	44.66	21.09	23.85
20-40	39.28	17.85	17.45	8.16	37.85	16.11	33.90	12.18	20.50
40-60	34.37	15.03	26.05	12.12	35.45	15.26	29.18	9.01	20.30
60-80	35.96	13.79	28.38	11.49	32.97	13.46	19.84	3.96	17.70
AVE	35.42	14.18	23.24	10.88	36.56	16.10	33.68	13.12	21.11

Field capacity and WC determine the range of soil availability water (Ritchie, 1981). The average range of availability water at 0-80 cm soil depth in NF, LT, and HT stands was 14.2-35.4, 16.1-36.6, and 13.1-33.7%, respectively, which was significantly higher than that in NT (10.8-23.2%). In brief, LT in these plantations can improve SWA status and approximate water conditions to those in the natural forest.

### The response of SOC and TN to SWA

A decrease in SOC and TN is often observed when soil surface cover is removed, depending on harvesting types, soil depths, stand density, clay content, and other factors (Nave et al., 2010; Hu et al., 2014; He et al., 2018). In this study, we found that TN and SOC decreased significantly with thinning (**Figure 5**). It could be expected due to greater exposure of topsoil to disturbance. Since the molecular complexity of SOC tends to be low in forest ecosystems, and the abundance of active substrates in the topsoil may stimulate microorganisms to respond more quickly following disturbance (Hu et al., 2014; He et al., 2018; Beillouin et al., 2022).

Bulk density, soil texture, and soil porosity are the objective indexes affecting soil water status. Among them, soil porosity and soil texture are significantly affected by SOC (Shan et al., 1998; Zhao and Wu, 2002). This is related to the loose and porous characteristics of SOC, which can promote soil porosity, water holding capacity, and water permeability (Adams, 1973; Rawls et al., 2004). In fact, Yu et al. (2021) and Huntington (2007) found that SOC indirectly affected SWA, which was consistent with our results.

### Implications for *P. crassifolia* forest management

In the past 40 years, China afforested a total of 3.63 × 10^8^ ha of land, with a forest tending and management area of 4.24 × 10^8^ ha, and forest cover has increased to 23.04% (Chen et al., 2019). However, a forest plantation is an ecosystem with high water consumption. Following large-scale afforestation in water-scarce areas, soil physicochemical properties of the topsoil and subsoil were changed, affecting soil water status. In addition, excessive planting density in the initial stage also aggravated the shortage of soil moisture and development of a dry soil layer. Proper stand management practices can improve soil water status by adjusting plantation density. Zhu et al. (2015) found that water deficit occurred in the 40-80 cm depth 29 years after afforestation at a planting density of 4458 trees ha^-1^, while light and heavy intensity thinning could significantly improve soil water storage at 0-80 cm depth. Heavy thinning intensity may provide an accommodative strategy for survival in water-scarce areas by reducing resources competition for the residual trees, but the long-term viability of this strategy is questionable (Sohn et al., 2016). In this study, we observed that different thinning intensity can significantly affect SWHC and SWA. Our results are consistent with the experimental results of He et al. (2012). We recommend that a soil dry layer caused by excessive planting density can be alleviated by thinning intensity of less than 40% or opening forest gaps. This can enhance forest water conservation, ensure sufficient water resources and ecological security, and realize the near-natural forest management and sustainable development of plantations.

## Conclusions

In this study, we used the Van Genuchten model to determine the response of SWA and SWHC in *P. crassifolia* to different forest management practices. Our results indicated that at low water-suction stage (0< pF ≤ 1.86), the topsoil (0-40 cm) and subsoil layer (40-80 cm) exhibited different SWHC characteristics. Among them, SWHC in topsoil in LT and HT stands was higher than that in NF, while SWHC in subsoil was lower in the NF stand. At medium and high-water suction (1.86< pF ≤ 2.52 and pF > 2.52), SWHC in NF and LT was higher than that in NT and HT stands. The changes in soil properties (BD, TP), particle-size distribution, and FC are the main reasons for the differences in SWA and SWHC across different stand management practices. In addition, the higher stand density reduced SWHC and SWA levels in NT, and a dry soil layer easily formed. In view of this, near-natural stand management, realized with 20% thinning intensity (light thinning) of plantations, can improve soil water status to a certain extent. In conclusion, our study provides empirical evidence for sustainable development of plantations in semi-dry areas and for alleviating soil water deficiency in QLM.

## Data availability statement

The original contributions presented in the study are included in the article/**Supplementary Material**. Further inquiries can be directed to the corresponding author.

## Author contributions

YG and XZ conceived and designed the experiments. YG participated in the field works. YG analyzed the data and wrote the original draft. ZH, XZ, LC, and JD revised the manuscript. All authors contributed to the article and approved the submitted version.

## Funding

This research was supported by the Strategic Priority Research Program of the Chinese Academy of Sciences (No. XDA23060301), the National Science Foundation of China (No. 42277481, 42207537), and the National Key Research and Development Program of China (No. 2019YFC0507403).

## Conflict of interest

The authors declare that the research was conducted in the absence of any commercial or financial relationships that could be construed as a potential conflict of interest.

## Publisher’s note

All claims expressed in this article are solely those of the authors and do not necessarily represent those of their affiliated organizations, or those of the publisher, the editors and the reviewers. Any product that may be evaluated in this article, or claim that may be made by its manufacturer, is not guaranteed or endorsed by the publisher.
