# Supplementary material for: Light thinning can improve soil water availability and water holding capacity of plantations in alpine mountains
Source: Front Plant Sci. 2022 Oct 12;13:1032057. doi: 10.3389/fpls.2022.1032057 (PMC9597321; doi:10.3389/fpls.2022.1032057)
Supplement: Supplementary file 1 [file DataSheet_1.docx]

Supplementary Material

## Supplementary Figures


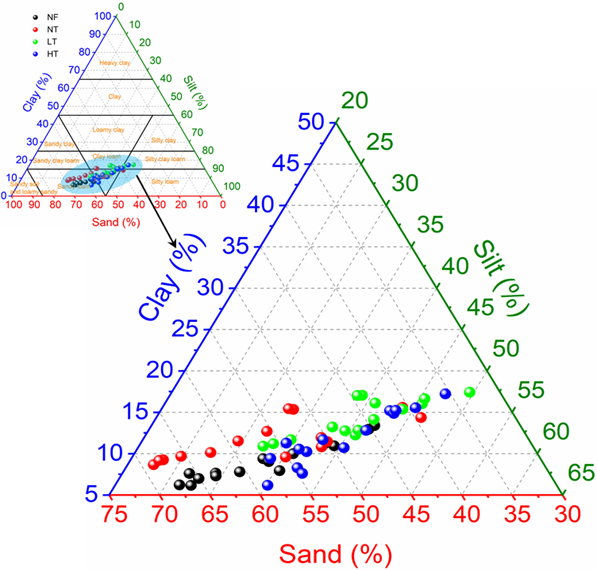


**Supplementary Figure 1.** Soil texture of the study plots. Black, red, green, and blue dots represent natural forest stand (NF), no thinning stand (NT), light thinning (LT), and heavy thinning (HT), respectively.


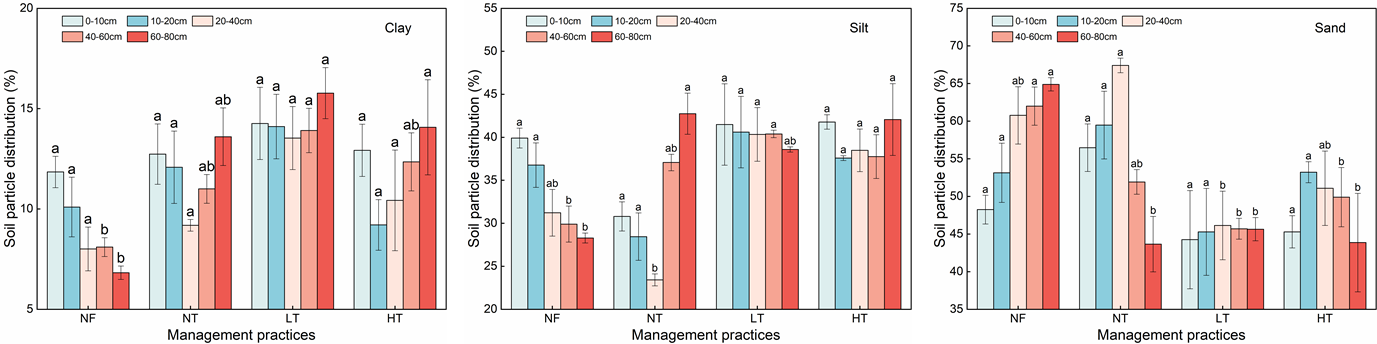


**Supplementary Figure 2.** Clay (< 0.002mm), silt (0.002-0.02mm), and sand (0.02-2mm) across different management practices and soil depths. NF, NT, LT, and HT represent forest stands with natural forest (reference stand), no thinning, light thinning (20% thinning intensity), and heavy thinning (40% thinning intensity), respectively. Different lowercase letters above the bars for same treatment indicate significant differences at *P*＜0.05 among soil depths.


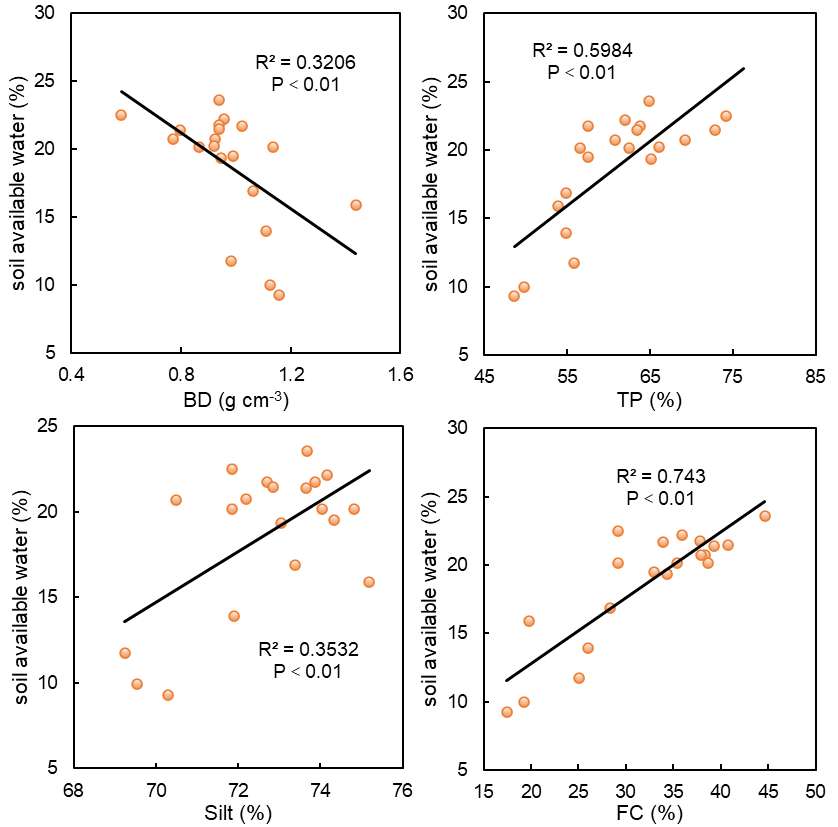


**Supplementary Figure 3.** Effects of four soil factors on soil available water.
